# Effects of intermittent overload doses of oral vitamin D_3_ on serum 25(OH)D concentrations and the incidence rates of fractures, falls, and mortality in elderly individuals: A systematic review and meta-analysis

**DOI:** 10.17305/bb.2024.10449

**Published:** 2024-10-01

**Authors:** Xiaoyang Tao, Wupeng Yang, Qinxin Zhang, Yongjiang Wang, Feng Gao, Yuehan Wang, Tingxin Zhang, Hao Liu, Jindong Chen

**Affiliations:** 1 Orthopedics Department, Ordos Central Hospital, Ordos, China; 2 Ordos No.1 Middle School, Ordos, China; 3 Ordos School of Clinical Medicine, Inner Mongolia Medical University, Ordos, China

**Keywords:** Vitamin D_3_, 25(OH)D, oral, load dose, intermittent, elderly

## Abstract

Vitamin D is commonly used to prevent and treat osteoporosis, with studies indicating its potential to reduce fractures, falls, and mortality. However, meta-analyses present inconsistent findings regarding its efficacy, particularly reflecting significant variability in data and outcomes related to various dosing regimens. In this meta-analysis, we assessed the impact of high-dose intermittent oral administration of vitamin D3 on serum 25(OH)D levels, fractures, falls, and mortality among elderly individuals. We included 14 randomized controlled trials (RCTs) and employed Review Manager 5.4 for statistical analysis. Our findings indicate that intermittent monthly administration of vitamin D3 (over 800 IU per day) significantly raised serum 25(OH)D levels at all timepoints after six months, maintaining levels above 75 nmol/L throughout the year. This regimen showed no increase in all-cause mortality, with a risk ratio (RR) (95% confidence interval [CI]) of 0.95 (0.87–1.04). Likewise, it did not significantly reduce the risks of falls and fractures, with RRs of 1.02 (0.98–1.05) and 0.95 (0.87–1.04), respectively. Although one-year intermittent administration significantly increased the concentration of 25(OH)D in serum, further research is needed to determine if this method would increase the incidence of falls. Therefore, it is not recommended at this stage due to the lack of demonstrated safety in additional relevant RCTs. This study had been registered at PROSPERO (CRD42022363229).

## Introduction

Vitamin D is not only widely used in the treatment of osteoporosis in middle-aged and elderly individuals but also has possible protective effects against cancer, infection, cardiovascular disease, and other diseases; therefore, it has wider indications [1–4]. It is generally considered that the current suitable concentration of 25(OH)D is between 30 and 11.94 ng/mL [5–7]. A lower dose (400 IU/day) has little effect on serum 25(OH)D concentrations, while 800 IU/day is the most commonly prescribed dosage [8]. In nursing home (NH) patients with severe 25(OH)D deficiency, an individually calculated cholecalciferol loading dose may be superior to a cholecalciferol 800 IU daily dose in rapidly normalizing vitamin D levels. This suggested that higher doses of vitamin D may be more rapid and effective in increasing serum 25(OH)D concentrations [9].

However, Dawson-Hughes and Harrisaso hypothesized that a 500,000 IU dose may trigger a “short-term protective” reaction in which CYP24 (25-hydroxyvitamin D-24-hydroxylase), the enzyme that catalyzes 1,25(OH)_2_D, is regulated, resulting in reduced serum and tissue levels of 1,25(OH)_2_D [10]. This hypothesis was consistent with results from an animal study [11]. The randomized controlled trial (RCT) conducted by Glendening [12] showed that there was no statistically significant difference in the mean serum 25(OH)D levels between the experimental and control groups after nine months. Therefore, it is necessary to investigate the effect and safety of high-dose, intermittent oral vitamin D_3_.

**Figure 1.**
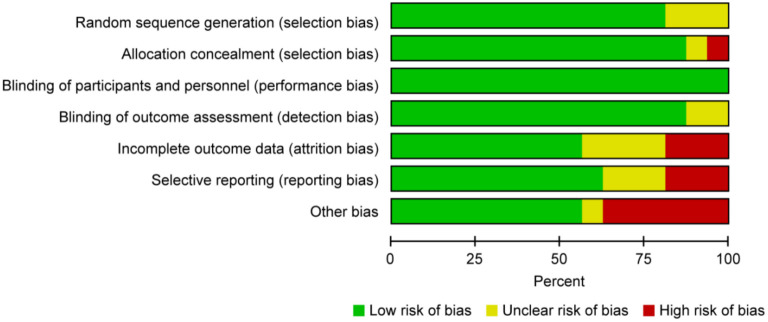
**Risk of bias assessment.** Judgments for each risk of bias item presented as percentages across all included studies.

In a previous meta-analysis, two different drugs, vitamin D_2_ and vitamin D_3_, were combined [13–17]. However, the results were inconsistent or heterogeneous. From a pharmacodynamic perspective, vitamin D_3_ has a greater ability to not only increase but also sustain higher serum 25(OH)D concentrations over time compared to vitamin D_2_ [18]. In addition, considering the reduced autonomy of elderly individuals, the compliance of daily administration was worse compared with intermittent oral administration, resulting in more cost [19], so a high-dose and intermittent oral vitamin D_3_ regimen may be more suitable for elderly individuals. Therefore, the purpose of this study was to investigate the clinical effects and safety profile of vitamin D_3_ with a meta-analysis conducted under the specific conditions of loading dose and intermittent oral administration.

## Materials and methods

### Meta-analysis of randomized controlled trials (RCTs)

RCTs were eligible for inclusion if they met the following criteria: 1) RCTs comparing vitamin D_3_ alone or in a combination with a placebo or a low dose (less than 400 IU per day); 2) The duration of the RCT was over 6 months, with the interval between doses more than 1 month, and each administration was a large dose (equivalent to more than 800 IU per day); 3) The average age of the participants was greater than 60 years old; 4) The mode of administration was limited to oral administration; and 5) The baseline serum 25(OH)D concentration of the included participants was greater than 30 nmol/L. The number of participants with one or more falls, fractures, and deaths were reported separately for the vitamin D treatment group and the control group.

Dosages were categorized as follows: 1) Low dose: Less than 400 IU per day; 2) Medium dose: Between 400 and 800 IU per day; and 3) High dose: Greater than 800 IU per day (and single dose greater than 40,000 IU).

Exclusion criteria were as follows: 1) RCTs with vitamin D_2_ or bisphosphonates; 2) RCTs that used active vitamin D, which requires monitoring for hypercalcemia, with much higher costs, thereby limiting their public health applicability; 3) Studies including patients with diseases that may lead to a significant decline in autonomy or motor stability, such as Parkinson’s disease, cerebral infarction, epilepsy, and other diseases; and 4) Studies that used intramuscular injections or intravenous administration.

### Data extraction and quality assessment

This study was carried out independently by two researchers between October 2022 and January 2023, according to the Preferred Reporting Items for Systematic Reviews and Meta-analysis (PRISMA) guidelines, and possible bias was assessed. PubMed, EMBASE, Cochrane Library, and other RCT databases were searched from database inception until January 30, 2023. We performed categorical analysis, heterogeneity checks, publication bias analysis, and subgroup analysis. The following data were extracted from the RCTs: year of publication, study design, sample size, duration of the intervention, percentage of women, number of total falls, fractures and deaths, serum 25(OH)D concentrations at different timepoints, and dosage and frequency of vitamin D administration. The authors of the included RCTs were contacted via e-mail for incomplete data. Some missing data were also derived from other previous analyses if the authors were unreachable. Quality assessment was performed by two independent researchers using the Cochrane Collaboration tool (Figure 1). We began our literature search in early 2024 while conducting the literature review. The risk of bias included in the literature was not high.

### Data synthesis and statistical analysis

The main outcome was the serum 25(OH)D concentration at different periods, followed by the incidence of fractures, falls, and death. Because the 25(OH)D concentration results of the combined RCTs were in different units (nmol/L or ng/mL), the results for the continuous variable were calculated using the standardized mean difference method. The Mantel–Haenszel method was used to calculate risk ratios (RRs) and their 95% confidence intervals (CIs). The *I*^2^ statistics or L’Abbe plots were used to assess the presence of heterogeneity, ranging from 0% to 100%. An *I*^2^ value greater than 70% suggested obvious heterogeneity and the need for a random-effect model. An *I*^2^ value between 40% and 70% represented moderate heterogeneity. A fixed-effect model was used for *I*^2^ values of less than 40%, which showed that heterogeneity could be disregarded. A funnel plot or Egger’s test was used to evaluate publication bias. A *P* value of less than 0.05 was considered to indicate statistical significance. Subgroup analysis was performed after grouping according to the duration of treatment. Fractures were defined as fractures of any part of the body except the vertebral body. The sensitivity analysis method refers to combining the remaining studies after deleting each study in each group to observe whether the results are consistent with the previous ones.

## Results

### Search results

**Figure 2.**
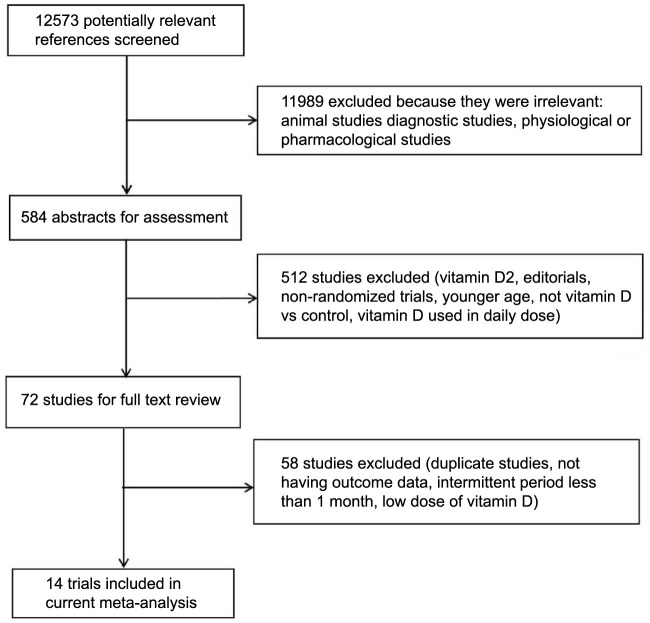
Study selection process flowchart.

An initial independent search of the electronic database identified 12,573 potentially relevant articles. After careful examination of the titles, 11,989 articles were excluded based on the inclusion criteria. Of the remaining 584 articles, 512 were excluded after carefully examining the abstract, mainly due to young age, vitamin D_2_ use, lack of a control group, etc. Out of the 72 articles read, 58 were excluded because of the lack of complete data results, noncompliance with the inclusion criteria listed above, and other reasons. Therefore, a total of 14 RCTs conducted between 2003 and 2022 were included in the final analysis, which contextualizes the varying follow-up durations from six months up to five years. Figure 2 provides a clear overview of the study selection process.

The main characteristics of the included studies are shown in Table 1. Eleven studies reported the concentration of 25(OH)D that was used for the main results, but a total of ten studies were included because the baseline concentrations of 25(OH)D were not provided in Trivedi’s experiment [20]. The analysis of death, falls, and fractures included six, nine, and six RCTs, respectively. The follow-up period ranged from six months to five years. The average age of participants ranged from 60 to 82 years, with the concentration of baseline 25(OH)D in most RCTs being less than 75 nmol/L.

**Table 1:** Characteristics of the included trials and participants

**Study**	**Treatment**	**Sex, female (%)**	**No. of participants**	**Mean age (years)**	**Post 25(OH)D (mean)**	**Observation** **(mean time point)**	**Outcome**
Malihi, 2019 [20]	100,000 IU monthly	59.1	170	65.9	61.9 nmol/L	3.3 years	VD^a^
	Placebo	57.1	163	65.9	61.6 nmol/L		
Khaw, 2017 [21]	Initial 200,000, then 100000 IU monthly	41	2539	65.9	64 nmol/L	3.4 years	Fall Fracture
	Placebo	43	2517	65.9	63 nmol/L		
Aspray, 2019 [22]	48,000 IU monthly	50.8	113	75.4	40 nmol/L	1 year	Fall VD
	12,000 IU monthly	45.2	112	74.6	40 nmol/L		
Waterhouse, 2021 [23]	60,000 IU monthly	46	1045	69.3	^b^	4.3 years	Fall VD
	Placebo	45.7	1048	69.3	^b^		
Trivedi, 2003 [24]	100,000 IU every 4 months	47.6	1345	76.1	Not mentioned	4 years	MortalityFallFracture
	Placebo	50	1341	75.4	Not mentioned		
Glendening, 2012 [12]	150,000 IU every 3 months	100	353	76.9	65.8 nmol/L	9 months	VD Fall Fracture
	Placebo	100	333	76.5	65.8 nmol/L		
Rachel, 2022 [25]	100000 IU monthly	45.9	10662	69.3	^c^	5.7 years	VDMortality
	Placebo	45.9	10653	69.3	^c^		
Sanders, 2010 [26]	50,0000 IU annually	100	1131	76	53 nmol/L	15 months	VD Mortality Fall Fracture
	Placebo	100	1125	76.1	45 nmol/L		
John, 2017 [27]	Initial 200,000, then 100000 IU monthly	40	71	64.5	62.1 nmol/L	1.1 years	VD
	Placebo	30	79	65.5	63.1 nmol/L		
Scragg, 2017 [28]	Initial 200,000, then 100,000 IU monthly	40.9	2558	65.9	26.5 ng/mL	3.3 years	VD Mortality
	Placebo	42.9	2550	65.6	26.5 ng/mL		
Scragg, 2019 [29]	initial 200,000, then 100,000 IU monthly	^d^	2558	65.9	64 nmol/L	3.3 years	Fall Fracture
	Placebo	^d^	2550	65.9	64 nmol/L		
Ginde, 2017 [30]	100,000 IU monthly	60	55	80	23 ng/mL	1 year	Mortality Fall Fracture
	Placebo	55.8	52	82	23 ng/mL		
Rake, 2020 [31]	100,000 IU monthly	53	392	^e^	52.4 nmol/L	2 years	VD Mortality
	Placebo	53	395	^e^	48.5 nmol/L		
Schwetz, 2017 [32]	Initial 540,000, then 90,000 IU monthly	54	153	62.2	13.9 ng/mL	6 months	VD Fall Fracture
	Placebo	51	136	60	13.7 ng/mL		

^a^25(OH)D concentration in serum; ^b^It was estimated that more than 76% of participants had vitamin D concentrations greater than 20 ng/mL (it was not specified precisely); ^c^It was estimated that more than 75% of participants had vitamin D concentrations greater than 20 ng/mL (it was not specified precisely); ^d^The values of the experimental group and the control group were not defined, and the average value of the two groups was 41.9%; ^e^No mean age was given, and the age range was 65 to 84; ^f^For the 25(OH)D concentration in serum, 2.5 nmol/L is equivalent to 1 ng/mL.

### Intermittent overload doses of oral vitamin D_3_ on serum 25(OH)D concentrations

**Figure 3.**
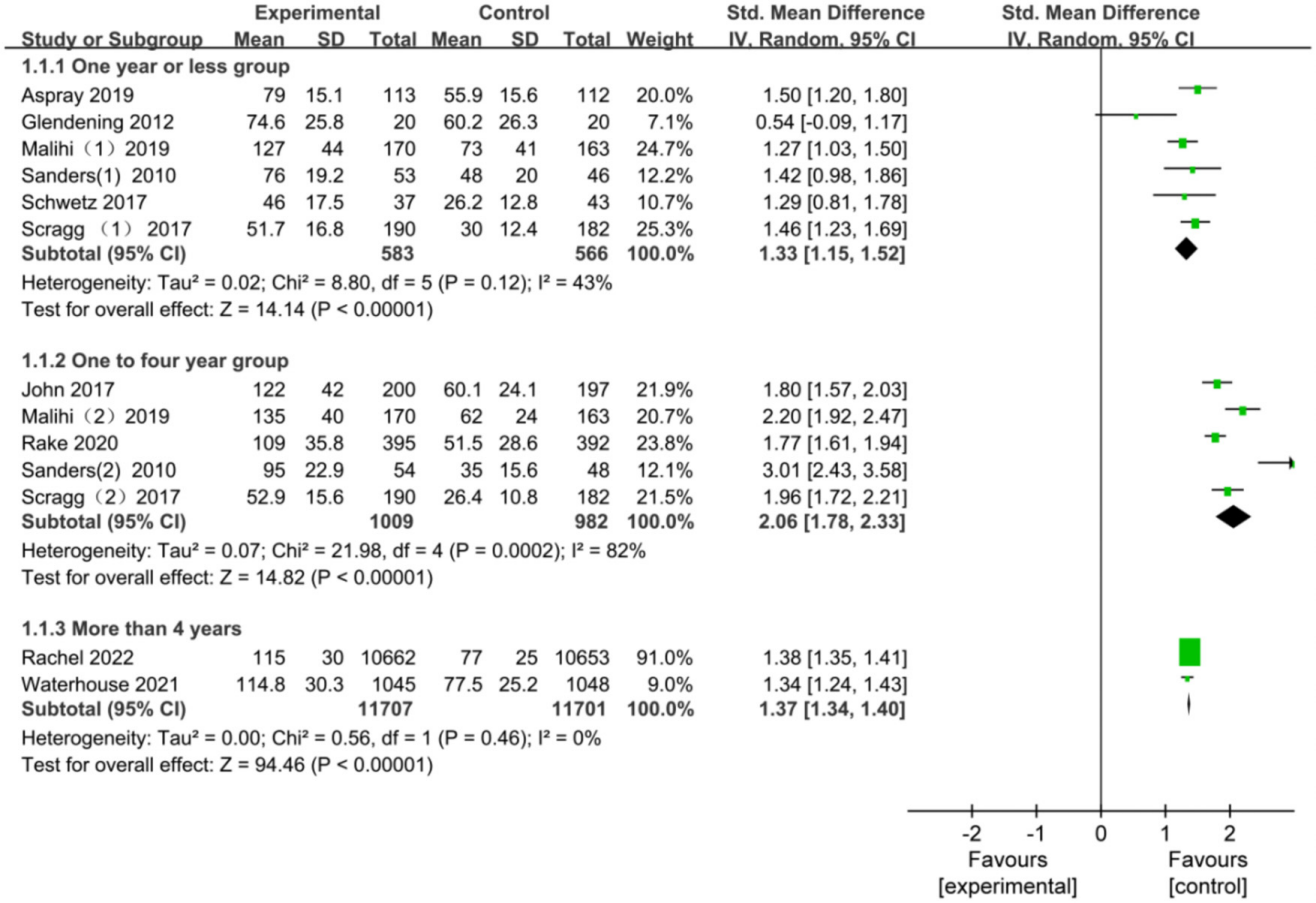
**Subgroup analysis by observation duration for vitamin D_3_ intervention.** Forest plot showing standardized mean differences (SMD) for 6 months to 1 year (SMD: 1.33, 95% CI: 1.15-1.52), 1-4 years (SMD: 2.06, 95% CI: 1.78-2.33), and over 4 years (SMD: 1.37, 95% CI: 1.34-1.40). Heterogeneity was moderate to high across subgroups. Egger’s test (0.7847) suggests no publication bias. CI: Confidence interval.

Based on the observation time, subgroups analysis included three subgroups: 6 months to 1 year, 1–4 years, and longer than four years, of which the standardized mean differences (95% CI) were 1.33 (1.15, 1.52), 2.06 (1.78, 2.33), and 1.37 (1.34, 1.40), respectively (Figure 3). The heterogeneity results of the group with less than one year were moderate (*I*^2^ ═ 43%), and subgroup analysis was performed. To further delineate the impact of administration frequency, we analyzed a subset of studies characterized by a one-month interval between vitamin D_3_ doses, termed one-month intermittent administration. This specific subgroup showed no significant heterogeneity (*I*^2^ ═ 0). There was significant heterogeneity in the second group (*I*^2^ ═ 82%) and moderate heterogeneity in the one-month intermittent subgroup (*I*^2^ ═ 61%), possibly due to the large difference in measurement times. The funnel plot suggested that the points on both sides were asymmetric; therefore, it was analyzed for publication bias by the quantitative method of Egger’s test (Egger ═ 0.7847), which indicated that there was no publication bias. In the sensitivity analysis, we combined the remaining study results after eliminating any study from the 1–2 years and the 1–4 years group, and it showed no change from the previous analysis, thus indicating that the results were stable.

**Figure 4.**
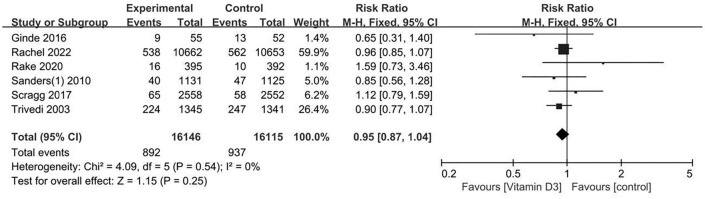
**Mortality risk in patients with intermittent high-dose vitamin D_3_.** This forest plot illustrates the risk ratio (RR) of mortality for patients treated with intermittent high-dose vitamin D_3_ versus controls. The pooled RR is 0.95 (95% CI: 0.87–1.04, *P* ═ 0.25), showing no significant effect on mortality. Data from 16,146 patients in the vitamin D group and 16,115 in the control group are included. CI: Confidence interval.

### Intermittent overload doses of oral vitamin D_3_ on mortality

The RR (95% CI) for mortality for patients treated with high-dose, intermittent vitamin D compared with the control was 0.95 (0.87–1.04) (Figure 4), which was not statistically significant (*P* ═ 0.25). A total of 892 of 16,146 participants (5.5%) randomized to the vitamin D group and 937 of 16,115 participants (5.8%) randomized to the placebo or no-intervention group died. The results remained robust after sensitivity analysis. We concluded that there was no publication bias by using Egger’s test (*P* ═ 0.7891).

### Intermittent overload doses of oral vitamin D_3_ on falls

**Figure 5.**
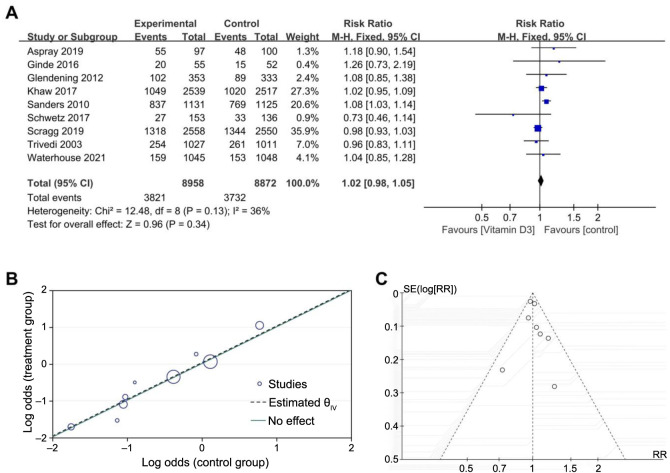
**Meta-analysis forest plot assessing the impact of intermittent overload doses of oral vitamin D_3_ on falls.** (A) The plot compares the risk ratios (RR) for falls between experimental groups treated with vitamin D_3_ and control groups across several studies; (B) The Labe plot illustrates the relationship between treatment effects and control event rates; (C) The funnel plot evaluates publication bias with symmetry around the effect size, suggesting no evidence of bias. CI: Confidence interval.

The RR (95% CI) for falls for patients treated with an overload dose and intermittent vitamin D compared with controls was 1.02 (0.98–1.05), without a significant difference (*P* ═ 0.34) (Figure 5A). The Labe diagram shows that the points were incompletely linearly distributed, and some points deviated far from the effect line, which suggested that heterogeneity was moderate for this outcome (*I*^2^ ═ 36%) (Figure 5B). After conducting a subgroup analysis based on the interval between drug administration, the heterogeneity disappeared (*I*^2^ ═ 0%) when the study by Sanders [21], which was administered intermittently for one year drug was excluded. The results remained robust after a sensitivity analysis using a funnel plot (Figure 5C) to analyze the publication bias, which suggested that the visible points were symmetrically distributed, presenting an inverted and incomplete symmetrical funnel shape. Based on Egger’s test, it was considered that there was no publication bias (*P* ═ 0.6508).

**Figure 6.**
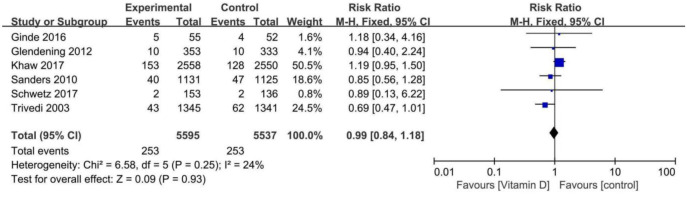
**Forest plots of the meta-analysis of fracture.** CI: Confidence interval.

### Intermittent overload doses of oral vitamin D_3_ on fracture

The RR (95% CI) for the hip frame in patients treated with overload dose and intermittent vitamin D_3_ compared with controls was 0.99 (0.84–1.18) (Figure 6), which was not statistically significant. The results suggested that intermittent overload doses of oral vitamin D_3_ increased the incidence of fracture, but the sensitivity analysis showed that the CI of Sanders’ experiment had changed significantly, while the statistical results and CI of other studies did not, so we deemed the results as unstable. However, a subsequent sensitivity analysis confirmed the stability of the results. Heterogeneity was observed for this outcome (*I*^2^ ═ 24%); but could be disregarded. Egger’s test was used to analyze the publication bias due to limited studies, and it showed no publication bias (*P* ═ 0.9127).

## Discussion

This meta-analysis has systematically evaluated the effects of intermittent administration of vitamin D on mortality, falls, and fractures, revealing nuanced differences between vitamin D2 and D3 supplements. We found that vitamin D3, when administered intermittently, may help reduce the incidence of fractures and falls without significantly impacting mortality rates. These outcomes underscore the potential benefits of vitamin D3 in enhancing bone health and preventing injury in at-risk populations. A previous review also suggested that vitamin D_2_ may not increase mortality [22]. However, Smith’s study [23] indicated that individuals receiving vitamin D_2_ had a significantly higher rate of fractures compared to the control group, raising concerns about the relative safety of this form of vitamin D. Our findings contribute to the ongoing debate by suggesting that vitamin D_2_ may not offer the same level of safety as vitamin D_3_, warranting further investigation into the differential impacts of these two compounds.

Decreased dose frequency has been identified as a factor associated with better responses to pharmacological therapy [24]. In addition, plasma 25(OH)D has a half-life estimated in terms of weeks rather than hours [25], so daily doses may not be required to maintain a steady vitamin D status. Most of the 25(OH)D concentrations reported by the RCTs included in this experiment were measured several days after administration; raising doubts about whether the concentration of 25(OH)D in serum could be significantly higher in the intervention group than that in the control group after 2–3 weeks. Armas et al. [26] chose a single bolus of 50,000 IU that showed a significantly greater AUC for cholecalciferol than for ergocalciferol, with serum 25(OH)D_2_ concentrations that fell rapidly back to baseline after only 14 days, whereas serum 25(OH)D_3_ concentrations peaked at the same time point and had not returned to baseline for the entire 28-day intervention. Sanders [21] used an annual intermittent drug administration period and performed tests at one and three months after the initial drug administration. Although the concentration in the third month was lower than the peak concentration in the first month, it was still significantly higher than that in the control group, with a concentration greater than 75 nmol/L, which was consistent with the conclusion demonstrated by Heaney et al. [27]: large doses of the vitamin are stored in fat and then slowly converted into serum 25(OH)D. However, daily administration had more advantages in the stability of the 25(OH)D concentration in serum.

Daily, weekly, and monthly vitamin D_3_ levels were compared in three trials. In one 4-month study of equivalent oral doses of vitamin D_3_ (600 IU/day, 4200 IU/week, and 18000 IU/month), the daily dose was the most effective and was the only dose that increased 25(OH)D concentrations [28]. However, in another experiment with a larger sample size, the comparison of three administration methods of 1500 IU daily, 10500 IU weekly, and 45000 IU every 28 days showed the same effectiveness results across all three regimens [29]. Essentially, the mode of administration for a higher dose may have different effects compared to a lower dose. In this meta-analysis, the dose for all RCTs in the experimental group was equivalent to more than 800 IU per day, potentially achieving higher serum 25(OH)D concentrations than those expected from less frequent, higher-dose regimens. In addition, Ilahi et al. [30] suggested that the dosing interval of intermittent dosing regimens should not be greater than 70 days to ensure that 25(OH)D levels do not decline below a target concentration of 70 nmol/L. Considering the adverse results of the annual administration analyzed previously, a monthly dosing interval may be more suitable.

According to the study of the group that was observed for more than 1 year, the concentration of 25(OH)D in serum was maintained between 44 and 56 ng/mL, which was much higher than the target concentration of 30 ng/mL [31]. Subsequently, issues, such as elevated serum and urine calcium, kidney stones, and other adverse events arose. However, the majority of disease-specific recommendations state consistently that the minimum serum 25(OH)D concentration should be 30 ng/mL, with an upper limit typically ranging between 50 and 60 ng/mL. Achieving and maintaining such values require regular vitamin D supplementation with doses of 3000–5000 IU/day [32]. It is generally assumed that large doses of vitamin D_3_ excreted through the kidneys can significantly increase the burden on the kidneys. Vieth et al. [33] conducted a 6-month safety and efficacy study and concluded that consumption of more than 4000 IU/day caused no harm and effectively raised 25(OH)D levels to “high-normal” concentrations (140 nmol/L) in practically all adults. In 2011, the Institute of Medicine’s report on dietary intake of vitamin D recommended an upper limit of 4000 IU/day and stated that doses up to 10,000 IU/day were safe. The studies included in this meta-analysis did not surpass the equivalent of 10,000 IU/day [34]. Malihi’s meta-analysis [35] suggested that intermittent administration of large doses (equivalent to more than 2800 IU/day) might increase the incidence rate of high serum calcium but not the risk of high urinary calcium or kidney stones. However, the inclusion criteria for that analysis did not limit age or the method of administration of vitamin D_2_ or vitamin D_3_; therefore, whether this conclusion is applicable to our meta-analysis, more RCTs that meet the aforementioned conditions are needed.

Vitamin D_3_ supplementation in appropriate doses is known to have a positive effect on fractures related to muscle function. However, very high doses of vitamin D can have a negative effect on muscle function due to a sudden increase in vitamin D receptor occupancy. Vitamin receptors are also present in the central nervous system [36], making it possible for falls to be affected as well. However, the exact amount of vitamin D_3_ administered that causes negative neuromuscular effects is unknown. Therefore, this may explain why there was no increase in the incidence of fractures and falls in the lower interdose group between January and April (receiving less than 200,000 IU as a single dose). In contrast, Sanders’ [21] study used a dose of 500,000 IU per administration and showed a significant increase in the incidence of falls. However, it should be noted that the results of the Schwetz [37] trial with a single dose of 540,000 IU showed no significant increase in the incidence of falls and fractures. Therefore, the negative effects of larger doses need to be verified in more RCTs.

In summary, we believed that this study had no obvious publication bias due to strict criteria for selecting RCTs and reducing heterogeneity after discussing the sources of heterogeneity in the analysis. Through sensitivity analysis, we came to a clear and convincing conclusion that oral vitamin D_3_ with more than 48,000 IU per month resulted in better compliance and was a more effective treatment regimen. However, this article does have some limitations. First, the intervention measures of the experimental group involved calcium, of which the preventive effect on fractures or falls was not analyzed in this article. Second, most included studies were conducted in regions far from the equator, namely, southeast Australia, New Zealand, the United Kingdom, and the United States. It is well known that the production of vitamin D is closely related to sunlight exposure, so this treatment may not be applicable to individuals living near the equator. The final conclusion cannot establish a secure upper limit for a single dose owing to insufficient evidence. However, a single dose of 200,000 IU was considered safe for administration.

## Conclusions

This analysis showed that receiving a high-dose dose (equivalent to more than 800 IU per day) of oral vitamin D_3_ every month for 1 year led to a significant increase in the concentration of 25(OH)D. Test results at any time after six months were above 75 nmol/L and this did not increase the incidence of fractures, falls, and deaths. Therefore, this treatment method can be promoted in middle-aged and elderly patients in high-latitude countries. Although one-year intermittent administration significantly increased the concentration of 25(OH)D in serum, whether the method would increase the incidence of falls requires further research and, thus, is not recommended due to the lack of safety demonstration with more relevant RCTs.

## Data Availability

Please contact the corresponding author for data requests.
